# Temperature-dependent carrier state mediated by H-NS promotes the long-term coexistence of *Y*. *pestis* and a phage in soil

**DOI:** 10.1371/journal.ppat.1011470

**Published:** 2023-06-22

**Authors:** Lihua Yang, Jing Wang, Shuguang Lu, Youhong Zhong, Kun Xiong, Xiaoxiao Liu, Bing Liu, Xiaoxue Wang, Peng Wang, Shuai Le

**Affiliations:** 1 Yunnan Key Laboratory for Zoonosis Control and Prevention, Yunnan Institute for Endemic Disease Control and Prevention, Dali, China; 2 Department of Microbiology, College of Basic Medical Sciences, Key Laboratory of Microbial Engineering Under the Educational Committee in Chongqing, Army Medical University, Chongqing, China; 3 Department of Frigidzone Medicine, College of High Altitude Military Medicine, Army Medical University, Chongqing, China; 4 Key Laboratory of Tropical Marine Bio-resources and Ecology, Guangdong Key Laboratory of Marine Materia Medica, RNAM Center for Marine Microbiology, South China Sea Institute of Oceanology, Chinese Academy of Sciences, Guangzhou, China; 5 Biobank, The First Affiliated Hospital of Xi’an Jiaotong University, Xi’an, China; Inserm, FRANCE

## Abstract

The study of carrier state phages challenged the canonical lytic-lysogenic binary, and carrier state appears to be ubiquitous and ecologically important. However, the mechanisms of the carrier state are not well elucidated due to the limited phage models. Herein, we reported phage HQ103, similar to *Escherichia coli* phage P2. In contrast to the temperate P2 phage, the HQ103 phage does not insert its genome into the bacterial chromosome and displays a dual behavior depending on the temperature. At 37°C, HQ103 lyses the host and forms clear plaques due to the truncation of repressor CI and mutation of promoter Pc. In contrast, HQ103 maintains a carrier state lifestyle with *Y*. *pestis* at an environmental temperature (21°C). Mechanistically, we found that the host-encoded histone-like nucleoid-structuring protein H-NS, which is highly expressed at 21°C to silence the Cox promoter Pe and inhibits the phage lytic cycle. Subsequently, the HQ103 carrier state *Y*. *pestis* could grow and co-exist with the phage in the soil at 21°C for one month. Thus, this study reveals a novel carrier state lifestyle of phage HQ103 due to the H-NS mediated xenogeneic silencing and demonstrates that the carrier state lifestyle could promote long-term phage-host coexist in nature.

## Author summary

The lifestyles of phages beyond the canonical lytic-lysogenic binary are being investigated in the natural environment. The alternative bacteriophage infection strategies mainly include pseudolysogeny and carrier state. Pseudolysogeny stands for a stalled state of development during which the phage genome does not replicate and is asymmetrically transmitted to a daughter cell. This is most often described in conditions of starvation. While the carrier state refers to a stable balance between phages and bacteria at a population level, caused by the persistence of both susceptible and resistant hosts. The ecological consequences of pseudolysogeny and carrier state had been suggested as maintaining both phage and bacteria under unfavorable conditions and promoting horizontal gene transfer. However, the study of the carrier state is very limited due to various technical challenges, including the lack of a stable carrier state model, and the molecular mechanism that maintains a carrier state lifestyle is not well understood. This study demonstrated an H-NS-mediated carrier state lifestyle between a lytic phage and *Yersinia pestis*, which promotes their coexistence in the soil at a cool temperature.

## Introduction

Phages are classified as virulent or temperate, and the virulent and temperate phages and their ecological impacts have been well investigated [1–4]. However, the canonical lytic-lysogenic binary has been challenged recently because pseudolysogeny and carrier state had become more ubiquitous than previously recognized and might play an important role in the ecology of phage-host interactions [5].

Pseudolysogeny is described as a state in which the phage episome resides in its host bacterium. The non-replicative and unintegrated phage genome is asymmetrically transferred to daughter cells during bacterial division, as observed in starved-induced growth-arrested bacteria [6]. The carrier state life cycle describes a population-level phenomenon, in which both sensitive and resistant bacteria exist and ensures the continuous propagation of the phage [5,7]. Thus, in the carrier state, phage progeny is continuously released by cell lysis, while no progeny is produced in the pseudolysogeny state. Moreover, recently, temperate phage P22 was found to maintain a carrier state with *Salmonella typhimurium*, dependent on the *pid* gene of P22. However, the molecular mechanism and implications of the carrier state are still elusive [8]. The evolutionary and ecological consequences of the carrier state have been acknowledged, such as maintaining both bacteria and phage under unfavorable conditions, promoting horizontal gene transfer between phage and host, and increasing the likelihood of recombination between phages within the infected host [5,6].

However, the study of carrier state is still very limited due to various technical challenges. First, the carrier state lifestyle is unstable and possibly temporal, and the cultivation conditions need to be substantially different. Second, different infection modes are present in the same population, such as the coexistence of susceptible and resistant hosts, making it difficult to detect the precise process of carrier state cycles. Briefly, the current carrier state phage models are not stable and ideal for studying the molecular mechanism and ecological impact. Investigating more comprehensive phage-host interactions under varying conditions is needed to increase our understanding of this field [9].

*Yersinia pestis* is responsible for the deadly plague, and its persistence in stable foci relies on the subtle balance between *Y*. *pestis*-contaminated soils, mammals exhibiting variable degrees of plague susceptibility, and fleas, which results in quiescent and epizootic periods of this zoonotic disease [10,11]. The *Y*. *pestis* phages have been isolated from various samples with significant diversity, including phages belonging to different viral families, and *Y*. *pestis* phages have been used for the diagnosis of *Y*. *pestis* [12–15]. However, the molecular interactions between *Y*. *pestis* and phage, and the ecological impact of phages on *Y*. *pestis* are poorly understood [15].

Herein, we report a *Y*. *pestis* phage HQ103, which maintains a carrier state with *Y*. *pestis* at environmental temperature (21°C) but turns lytic at warm temperature (37°C) to lyse bacteria. Moreover, the HQ103 co-exists with *Y*. *pestis* in the soil at 21°C for over one month. Thus, this study not only reveals a novel temperature-dependent carrier state phage lifestyle but also suggests that carrier state could promote the coexistence of phage and bacterium in nature.

## Materials and methods

### Bacterial strains, phages and culture conditions

The bacterial strains, phages and plasmids used in this work are listed in Table 1. *Y*. *pestis* strain EV76 [16] was grown on Lysogeny Broth (LB) at 21°C or 37°C. When required, ampicillin (100μg/ml), and kanamycin (50μg/ml) were used. Phage HQ103 was isolated from the feces of a wild healthy rat, which was captured in the plague foci in Heqing County, Yunnan Province, China. The phage HQ103 was cultured in LB in the presence of EV76 at 37°C. Experiments with wild-type *Y*. *pestis* were performed in a biosafety level 3 (BSL3) lab at the Yunnan Institute for Endemic Disease Control and Prevention.

**Table 1:** Strains and plasmids used in this study.

Strains and plasmids	Description	Source
**Strains**		
EV76 EV76/p*hns* EV76/p*recA* EV76/p*lexA* EV76/p*CI* EV76/p*CI*_*EC12*_ EV76/p*int* EV76/p*cox* EV76/pPe EV76/pPc *EV76/p*Pe*-hns*	*Yersinia pestis* strain Overexpression of *hns* in EV76 Overexpression of *recA* in EV76 Overexpression of *lexA* in EV76 Overexpression of *CI* in EV76 Overexpression of *CI*_*EC12*_ in EV76 Overexpression of *int* in EV76 Overexpression of *cox* in EV76 EV76 strain with the Pe promoter-GFP reporter plasmid EV76 strain with the Pc promoter-GFP reporter plasmid EV76 strain with the Pe promoter-GFP reporter plasmid and *hns* is overexpressed	[16] This study This study This study This study This study This study This study This study This study This study
**phages**		
HQ103 HQ103Δ*CI* HQ103Δ*int* HQ103Δcox	*Yersinia pestis* phage Knock out *CI* in phage HQ103 Knock out int in phage HQ103 Knock out *cox* in phage HQ103	This study This study This study This study
**plasmids**		
pBAD24 pET28a pTCPLS PLI50-10S	Plasmid for over-expression of gene in EV76 Plasmid for protein expression and purification Plasmid with CRISRPR-cas9 for gene knockout in EV76 Plasmid for gene promoter activity detection	[31] Novogene This study [32]

### Transmission Electron Microscopy (TEM)

The morphology of the purified phage HQ103 was observed using transmission electron microscopy (TEM) as previously described [17]. The phage solution was dropped onto carbon-coated copper grids for 15 min and negatively stained with 2% phosphotungstic acid (pH 7.0) for 30 s. Then, the phage particles were observed using TEM and the size of the phage was estimated based on the average of 4 phages.

### Plaque assay and efficiency of plating (EOP) assay

A phage plaque assay was performed by spotting phages on bacterial soft agar overlays. Briefly, 100 μL of log phase bacterial culture, which was cultured to an OD600 of 0.2, was mixed with 100 μL of diluted phage and 6 mL of 0.4% LB agar, overlaid onto LB agar plates. Then, the plates were incubated for 18 h at 37°C or 21°C.

Twenty wild-type *Y*. *pestis* strains and six wild-type strains of *Y*. *pseudotuberculosis* preserved in our lab were used to determine the host range of phage HQ103 using the EOP assays incubated under 37°C or 21°C, respectively. The strain was sensitive to phage HQ103 if it forms plaques (S1 Table). The EOP of phages on different bacteria (Table 1) was determined as previously described [18]. Briefly, 5 μl of serial 10-fold dilutions of a phage solution were spotted on double-layer agar plates that were premixed with a host strain and incubated the plates for 18 h at 37°C or 21°C. The number of plaques observed after overnight incubation was compared to the number obtained on sensitive strain EV76 incubated at 37°C.

### Phage and bacteria genome sequencing and analysis

The genome of the phage particles was extracted, as previously described [19], and the genome was sequenced using Illumina Hiseq 2500 platform (~1 Gbp/sample). Sequencing data were pre-processed with Fastp v.20.1 [20] and assembled using SPAdes v3.15.2 [21] with parameters “-k 127—isolate”. Next, the assembly graphs from SPAdes were visualized with Bandage v0.8.1 [22], and the sequences were manually chosen and exported. Then, the phage termini were predicted with PhageTerm v1.0.12 [23]. Finally, the phage genomes were annotated with Bakta v1.2.4 [24].

The DNA of EV76 was extracted using the Cetyltrimethyl Ammonium Bromide (CTAB) method and sequenced using the Pacific Biosciences platform and the Illumina Miseq platform. After adapter contamination removal and data filtering, data assembly proceeded by using AdapterRemoval [25] and SOAPec [26]. The filtered reads were assembled by SPAdes [21] and the scaffolds and contigs were constructed by A5-miseq [27]. The Pacbio platform sequencing data was assembled by Canu [28]. Subsequently, all assembled results were integrated to generate a complete sequence after the rectification by pilon software [29].

The genome of EV76::HQ103 was sequenced using Illumina Hiseq 2500 platform (~1 Gbp/sample) and mapped using the EV76 genome as a reference. The integration site of phages was manually checked through the sequencing data of EV76::HQ103 using the BLAST+ tools in Bandage [30], which failed to identify the integration site. Then, the reads were assembled de novo, and the phage genome was identified as an independent element.

### Identification of the HQ103 lysogen by PCR

Phage HQ103 and bacterium EV76 were mixed with a ratio of 10:1, inoculated onto an LB agar plate, and incubated at 21°C until the colonies formed. Then, a single colony was isolated for PCR using primers HQ103-1F/R (S2 Table) to detect the presence of HQ103. Then, the HQ103-positive colony was streaked on an LB agar plate followed by incubation at 21°C for 72 h, referred to as 1^st^ generation progenies. Fourteen to twenty 1^st^ generation colonies were randomly selected for PCR using primers HQ103-1F/R to detect the presence of HQ103 and calculate the positive rate. Then, the HQ103 positive 1^st^ generation colony was streaked on an LB agar plate again, and the colonies formed after incubation at 21°C were referred to as 2^nd^ generation progenies, and PCR was used to determine the HQ103 positive rate. Three biological replicates were performed to calculate the positive rate.

### Bacteriophage adsorption assay

*Y*. *pestis* EV76 was cultured at 21°C or 37°C until OD_600_ reached 0.5, and phage HQ103 was added at a multiplicity of infection (MOI) of 0.01, and the mixture was maintained at 21°C and 37°C for 15 minutes, then centrifuged at 16,000 g for 1 minute. The negative control group was performed by mixing phages with LB instead of bacterial culture. Then, the phage titer in the supernatant (t2) and the original phage stock (t1) was determined using double-agar plating assays. The phage adsorption rate was calculated as (t1 –t2)/t1. The means and standard deviations are calculated from three biological replicates.

### RNA-SEQ

The host bacterium EV76 was cultured at 21°C or 37°C with shaking until OD600 reached 0.5. The bacteria were collected by centrifugation, immediately frozen with liquid nitrogen, and stored at -80°C. Three biological repeats were performed. RNA extraction and RNA-seq analysis were performed as described earlier [26]. Briefly, the SV Total RNA Isolation System (Promega, USA) was used to isolate the total RNA, and the RNA quality and quantity were checked using Bioanalyzer (Agilent, USA) and RNA 6000 Nano kit (Anilent, USA). Then, rRNA was removed using the Ribo-Zero rRNA removal kit, then the cDNA libraries were constructed and sequenced on an Illumina HiSeq 2500 sequencer (Illumina, USA) using the 2 × 150 bp paired-end mode. Gene expression changes were measured by the Reads Per Kilobase Per Million Read (RPKM) and the false discovery rate (FDR) (q value). Differentially expressed genes (DEGs) between the 21°C or 37°C culture groups were calculated by DESeq, and genes with a fold change value of >2 and a q value of 0.05 were determined as DEGs.

### Quantitative real-time reverse-transcription PCR (qRT-PCR)

The bacteria were cultured at 21°C and 37°C, and were collected by centrifugation at a turbidity of 0.5 at 600 nm. Total RNA was extracted and reverse-transcribed, as described previously [26]. Then, qRT-PCR was performed using SYBR Premix Ex Taq II kit (TaKaRa Bio, China), and the primer sets provided in S2 Table. The level of 16s rRNA transcript was used to normalize the gene expression data [31].

To determine the expression of phage genes, phages were added with an MOI of 10 into the bacterial culture, and after 2min of infection, the bacteria were collected by centrifugation for 1 min and the total RNA was extracted immediately. The relative expression levels of phage genes were detected by qRT-PCR with primers listed in S2 Table and the 16S rRNA gene was used to normalize the gene expression data. Three biological repeats were conducted for each experiment.

### Overexpression of genes in EV76

DNA sequences of interest (*CI*, *cox*, *int*, and 294-bp of *CI*_EC12_) were amplified by PCR using phage DNA or chemically synthesized *CI*_*EC12*_ DNA, and the linear pBAD24 vector was also generated by PCR using plasmid DNA [32]. The primer sets are described in S2 Table. Next, the amplicons of *CI*, *cox*, *int*, and *CI*_EC12_ were cloned into the linear vectors using the pEASY-Uni Seamless Cloning and assembly kit (Transgen, China). The genes of interest (*hns*, *recA*, and *lexA*) were amplified by PCR using *Y*. *pestis* DNA with primers listed in S2 Table. The amplicons of *hns*, *recA*, and *lexA* were digested with EcoRI/XbaI, NcoI/HindIII and EcoRI/HindIII, respectively. Then, the fragments were cloned into the EcoRI/XbaI, NcoI/HindIII and EcoRI/HindIII digested pBAD24, respectively. All recombinant plasmids were then transformed into *Escherichia coli* Trans1-T1 by electroporation. After sequence verification of the cloned sequences, the recombinant plasmids were transferred into the EV76 strain by electroporation. Lastly, the constructed strains were inoculated into the culture media supplemented with 0.2% of arabinose to induce the expression of the cloned genes.

### Purification of His-tagged H-NS protein

The *hns* gene fragment was amplified by PCR with primers listed in S2 Table, and ligated to the protein expression vector pET28a, and transformed into *E*. *coli* BL21(DE3). The verified expression strain was cultured in the presence of kanamycin until the turbidity reached OD600 of 0.8. Then, 1mM isopropyl β-D-1-thiogalactopyranoside (IPTG) was used to induce the protein expression at 37°C for 5 h. The cells were collected, resuspended in lysis buffer, and disrupted by a French Press (Thermo Fisher Scientific). Finally, the protein was purified using Ni-NTA resin (Qiagen, Duesseldorf, Germany) according to the manufacturer’s protocol and validated on SDS-PAGE.

### Electrophoretic mobility shift assays (EMSA)

EMSA was performed as previously described [33,34]. The Pc and Pe promoter regions were amplified by PCR from the phage genome with biotin-labeled primer pairs listed in S2 Table. A fragment of *psaE* that could bind to H-NS was selected as a specific competitive probe, and a fragment of 16s rRNA gene that could not bind to H-NS was used as a non-specific competitive probe [34]. Moreover, Bovine serum albumin (BSA) was used as a negative control. The *psaE* and 16S regions were amplified from the genome of EV76. The PCR product was purified and 200 ng of PCR fragment was incubated with 1.6μg of H-NS in a 20-μl reaction mixture containing 10 mM HEPES (pH 7.6), 1 mM EDTA, 2 mM dithiothreitol, 50 mM KCl, 0.05% (vol/vol) Triton X-100, and 6% (vol/vol) glycerol. Binding reactions were performed for 30 min at room temperature. H-NS was replaced by double-distilled water in the blank control group, and by BSA (1.6 μg) in the negative control group. In the competing probe group, 1.6 μg of H-NS and 200ng of probe *psaE* or *16s* were added, and then 200ng of biotin-labeled target probe Pe-Bio or Pc-Bio was added, and the reaction was performed for 30 minutes at room temperature. Then, the mixtures were separated on 6% (mass/vol) native polyacrylamide gel electrophoresis in 0.5× TBE (Tris-boric acid-EDTA) buffer at 80 V for 2 h at 4°C. After electrophoresis, the film was transferred on the ice at 380 mA for 1h, and the nylon film was cross-linked in the UV-light cross-linker (120mJ, 120s). Then Chemical Nucleic Acid Detection Module kit (Thermo Fisher Scientific) was used to detect the image.

### Reporter system

This study used a pLI50-10S vector [33] with a GFP UV reporter gene to clone the Pe and Pc promoters, respectively. First, the promoter region was PCR amplified with primers RE-pe-F/R from the phage genome, and primers RE-pe-P-F/R were used to amplify the plasmid scaffold. Then, the two fragments were ligated by Seamless Cloning and Assembly Kit and transferred into *E*.*coli* Trans1-T1. To construct a Pe promoter-GFP reporter when *hns* is overexpressed, the Pe-GFP region was amplified by PCR using primer HNS-GFP-gF/R, and then *hns* and plasmid scaffolders were amplified by primers HNS-GFP-hF/R and HNS-GFP-pF/R, respectively. Three fragments were ligated using the Seamless Cloning and Assembly Kit and transferred into *E*. *coli* Trans1-T1. The recombinant plasmid was verified by Sanger sequencing and transferred to the host bacterium EV76, and the positive clone was screened. Then, EV76/pPe, EV76/pPc and EV76/pPe*-hns* were cultured at 21°C or 37°C until the OD600 reached 0.5. Then, the images were acquired using fluorescence microscopy (×1000 magnification). Moreover, the same number of bacteria was used to measure the fluorescence intensity. Three biological replicates were performed on different dates.

### Knock out phage genes using the CRISPR system

The CRISPR-Cas9 plasmid pPTCS was constructed by inserting the Cas9 and spacer into pHerdB20T (S1 Fig). To knock out *CI* in phage HQ103, the spacer was generated by annealing primersΔCI-G1F/R (S2 Table) and ligated into the Eco31I digested pPTCS. To construct the recombination template, primersΔCI-LA-F/R and ΔCI-RA-F/R were used to amplify a 500 bp fragment before and after the *CI* gene, respectively, and ligated into the multiple cloning site of pPTCS by Gibson Assembly. Then, the plasmid was transferred into EV76. The strain EV76/pTCPLS-ΔcoxG3D was then infected with 10^5^ pfu of phages and plaque assay was used to select the survivors. The mutant phage was verified by PCR followed by Sanger sequencing. Knock out of *CI*, *Int* or *cox* in phage HQ103 was performed with similar technology with primers listed in S2 Table and the process is illustrated in S1 Fig

### Bacteria-phage co-evolution experiment in soil

The experiment was conducted at 21°C or 37°C with modifications from the previous protocol [35]. EV76, HQ103, or EV76::HQ103 lysogen were inoculated into the autoclaved soil, which was collected from a mountain in Heqing County, Yunnan Province, to a final density of ~10^5^ CFU/g, ~10^3^ PFU/g, and ~10^5^ CFU/g, respectively. Three biological replicates were performed for each group. The PFU was measured at the given time points using plaque assay under 37°C, and the CFU was determined by calculating the colonies formed under 21°C.

### Statistical analyses

Student’s t-test was used to compare two-group data, and a *P* value < 0.05 was considered as statistically significant.

## Results

### Identification and characterization of phage HQ103 lysis *Y*. *pestis*

**Fig 1:**
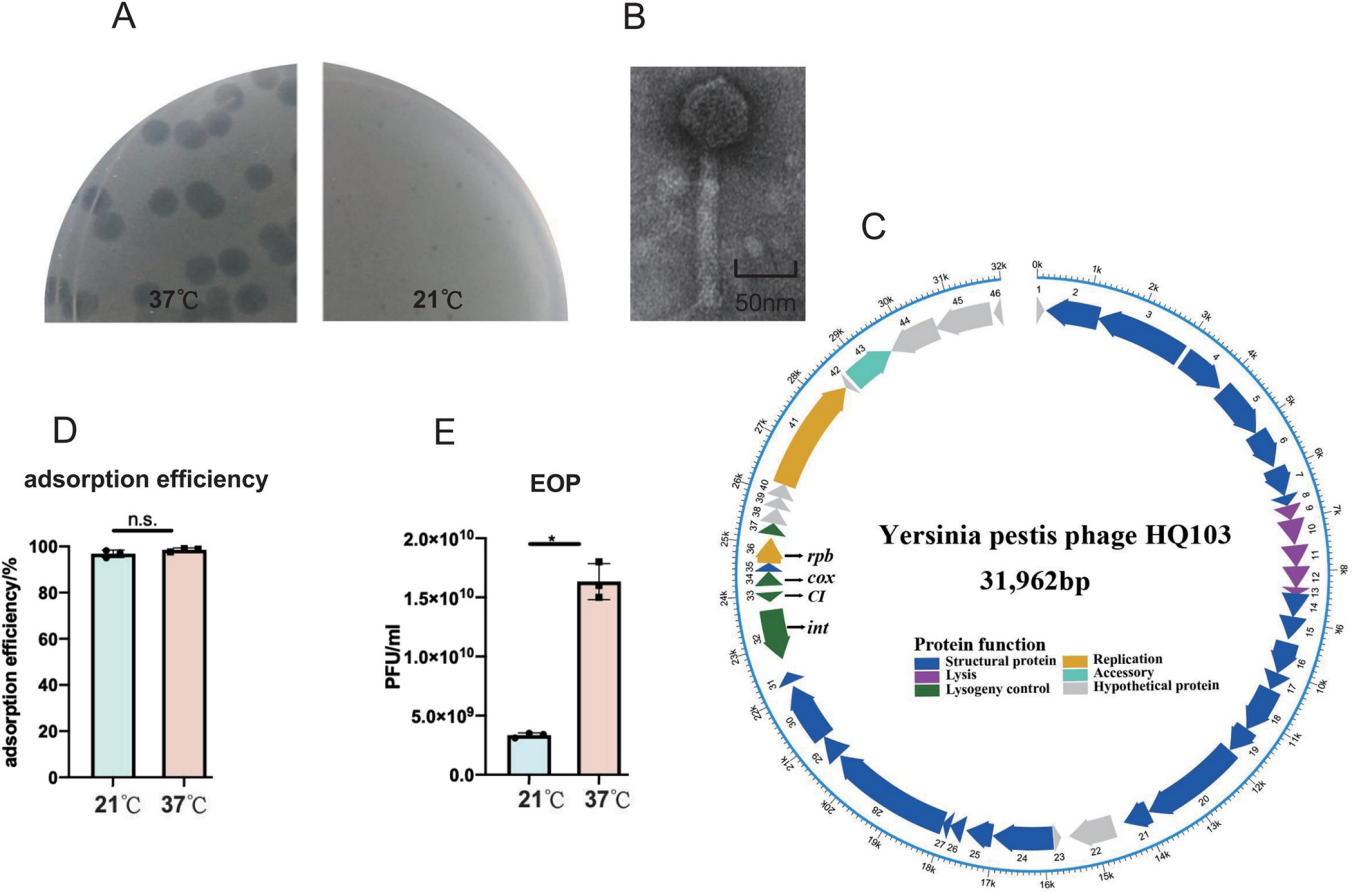
Characterization of *Y*. *pestis* phage HQ103. **(A)** Phage HQ103 forms large and clear plaques on *Y*. *pestis* at 37°C and forms small and blurred plaques at 21°C. **(B)** The transmission electron microscopy image of phage HQ103 indicates it as a *Peduoviridae* phage. **(C)** The genomic map of HQ103 and its functional modules are labeled, including a lytic-lysogenic regulon. **(D)** Phage HQ103 adsorbs to the host efficiently at both 21°C and 37°C. **(E)** The efficiency of plating (EOP) of HQ103 against *Y*. *pestis* strain EV76 at 21°C is only 20.41% as that of 37°C. The asterisks mark *P*-value of < 0.05 as calculated by Student’s t-test, and n.s means no statistical difference.

A plaque-forming lytic phage that we named HQ103 was isolated using *Y*. *pestis* strain EV76 as a host from the feces of a rat, which was captured in Heqin County, Yunnan Province in China (Fig 1A). Transmission electron microscopy (TEM) reveals that it has an icosahedral capsid with ~60 nm in diameter and a ~140 nm contractile tail (Fig 1B). It forms clear plaques on all 20 *Y*. *pestis* strains we tested at 37°C but could not lyse the 6 *Y*. *pseudotuberculosis* strains (S1 Table). However, the genomic sequence reveals that it shares 96.58% identity with the *E*. *coli* lysogenic phage P2 [36], and therefore phage HQ103 belongs to the *Peduoviridae* family [37]. The genome of HQ103 is 31,962 bp in length with a predicted 19-bp 5’ single-stranded cohesive ends (GTGCTTTCCCCGCCTCGCC) by PhageTerm v1.0.12 (Fig 1C). To be noted, HQ103 also has a similar lytic-lysogenic switch region as that of phage P2, including a lysogenic module (integrase, repressor) and lytic module (Cox). However, HQ103 forms clear plaques at 37°C, unlike its *E*. *coli* counterpart. Interestingly, we found that temperature is a key factor for its lytic cycle as it only forms small blurry plaques at 21°C (Fig 1A). Thus, this phage presents genes classically encountered in temperate phage genomes and its efficiency of making plaque is temperature-dependent.

### Phage HQ103 is in a carrier state at 21°C

To further characterize the temperature effect on the lytic ability of HQ103, we tested its absorption efficiency and the efficiency of plating (EOP) for EV76 at 21 and 37°C. The results suggested that while there was no difference between the adsorption rates at the two temperatures (Fig 1D), the EOP dropped significantly at 21°C to only 20.41% of that at 37°C (Fig 1E). Thus, the switch between lytic-lysogenic lifestyle is likely to be regulated via the temperature sensing pathway of the host.

**Fig 2:**
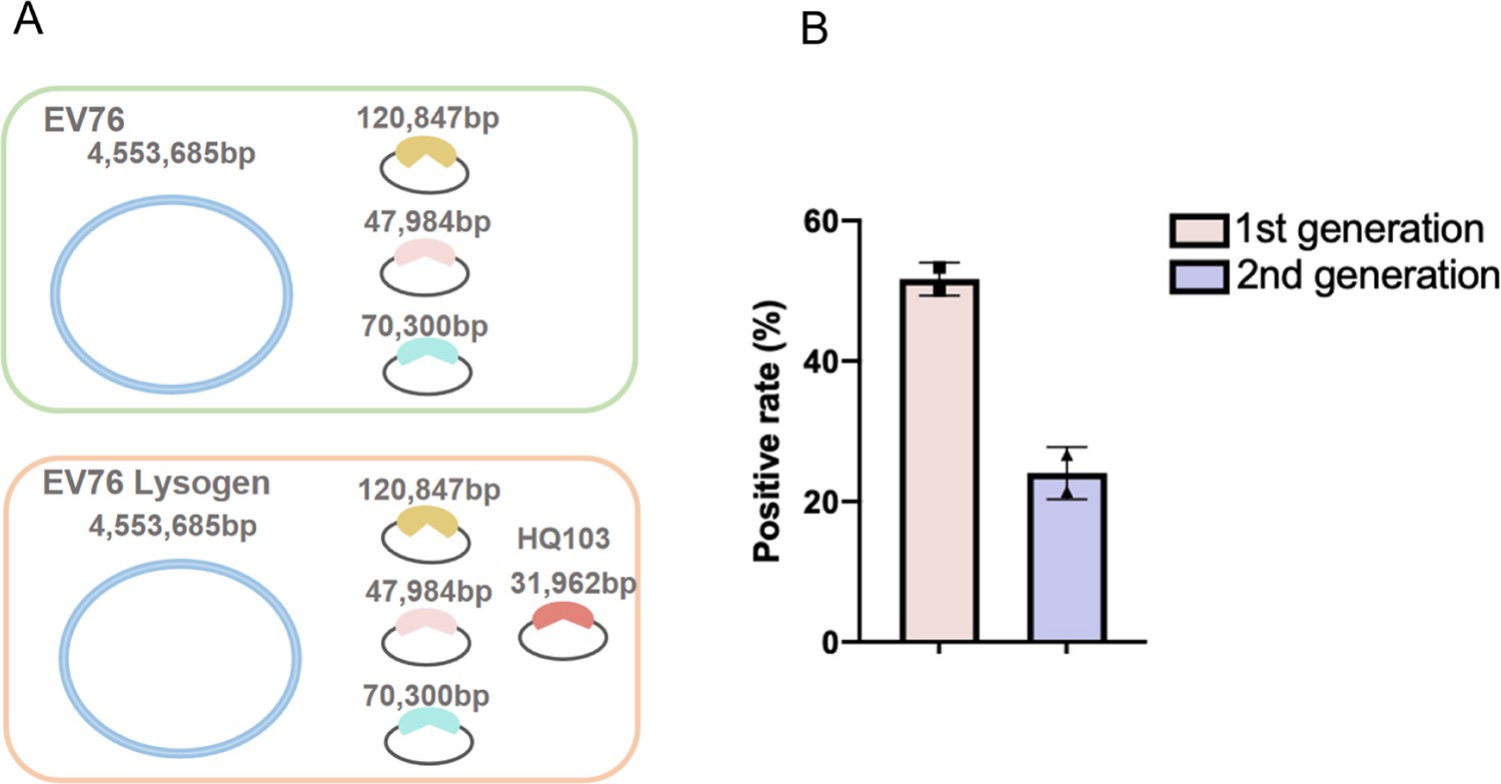
Phage HQ103 is in carrier state at 21°C. **(A)** Comparative genomic analysis of *Y*. *pestis* strain EV76 and HQ103 carrier state EV76 reveals that HQ103 exists as a circular element instead of integrated into the bacterial genome. **(B)** The positive rate of the first- and second-generation colonies that contain phage HQ103 was 51.67±1.67% and 24.05±2.62%, respectively.

To determine the integration site of HQ103, we first isolated the phage HQ103 lysogens of EV76, which we refer to as EV76::HQ103, at a temperature of 21°C. We confirmed the presence of the phage genome through PCR analysis, sent both EV76 and EV76::HQ103 for genome sequencing. However, as the previously reported genome of EV76 (NCBI access number SAMN02403074) was incomplete, we utilized a combination of third-generation sequencing and next-generation sequencing to map the complete genome of EV76, which consists of a genome and three large plasmids (Fig 2A). We then mapped the genome of EV76::HQ103 through next-generation sequencing, which indicated that HQ103 was maintained as an independent element. We observed no similar sequences between the genome of HQ103 and EV76, and no phage integration site was detected.

After subculturing the HQ103 lysogens, we observed that 51.67±1.67% of the progenies were lysogenic. This suggests that nearly half of the bacteria population lost phage during replication. We then subcultured the positive colony and found that only 24.05±2.62% of the second-generation colonies contained HQ103 (Fig 2B). Based on these findings, we conclude that HQ103 may not be able to integrate into the genome of EV76, and HQ103 is unable to replicate efficiently in this state. Furthermore, HQ103 appears to divide asymmetrically during bacterial division, indicating a carrier state lifestyle in EV76 at 21°C.

### The carrier state is maintained by H-NS at 21°C

The impact of temperature on the phage lifestyle inside *Y*. *pestis* was then examined using RNA-seq to identify potential host factors that affect the phage lifestyle switch. The result showed that 1,387 genes were differentially regulated under the two temperatures, highlighting the significant impact of temperature on *Y*. *pestis* [10,38]. The bacterial pathogen detects a temperature shift as a cue to alternate between their vertebrate and invertebrate hosts, inducing adaptive gene expression and phenotypic changes to successfully infect the vertebrate host [39,40]. The study also revealed that 15.5% of host genes were up-regulated at 37°C, while 15.8% were down-regulated, indicating changes in cellular processes, environmental information processing, metabolism, and other factors. Notably, the virulence genes, such as the type III and type II secretion system, and the pilus and flagellar assembly proteins, were highly expressed at 37°C, which is the body temperature of mammals, and were depressed at 21°C, which represents the environmental temperature (S3 Table).

**Fig 3:**
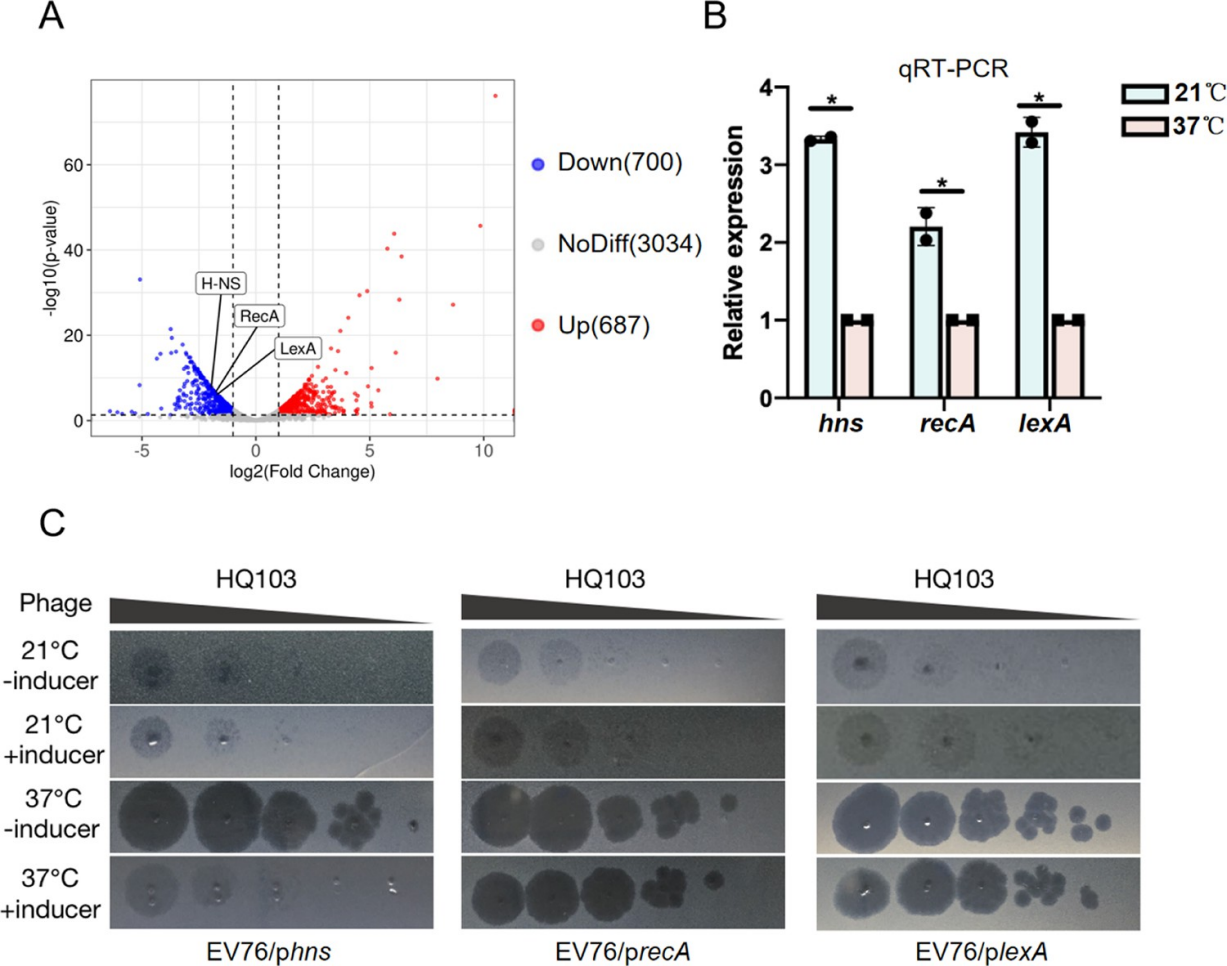
H-NS is downregulated at 37°C and could repress the phage lytic cycle. **(A)** The differentially expressed genes of EV76 at 21°C and 37°C were detected by RNA-seq. **(B)** RT-QPCR validation of the expression of *hns*, *recA* and *lexA* at 21°C and 37°C. The asterisks mark *P*-value of < 0.05 as calculated by Student’s t-test. **(C)** Over-expression of *hns*, but nor *recA* or *lexA*, could inhibit the lytic cycle of HQ103 at 37°C.

Among proteins whose genes were down-regulated, the histone-like DNA-binding protein H-NS is known to respond to temperature fluctuations and repress gene expression by binding to AT-rich regions [41–43]. In EV76, the *hns* gene is down-regulated at 37°C (Fig 3A). To investigate this further, we measured the mRNA expression of *hns* at 21°C and 37°C, which showed that *hns* is expressed approximately 3.3-fold more at 21°C than at 37°C (Fig 3B). To explore the role of *hns* in the carrer state-lytic switch of phage HQ103, we cloned the *hns* gene into a plasmid with an arabinose inducible promoter and transferred it into *Y*. *pestis* strain EV76. When grown at 37°C, HQ103 forms clear plaques on EV76/p*hns*, but forms blurred plaques on the same strain supplemented with arabinose, indicating that the temperature-dependent expression of *hns* is likely to be responsible for the switch (Fig 3C). Our attempts to use CRISPR-cas-based gene knockout technique to delete *hns* gene from EV76 were unsuccessful, consistent with a previous study, indicating that *hns* is an essential gene for the *Yersinia* genus [44].

The SOS response is another common pathway for activating prophages, including P2-like phages [45,46]. Although we observed overexpression of *recA* and *lexA* at 21°C (Fig 3B), their overexpression did not lead to blurred plaques (Fig 3C). This rules out the involvement SOS pathway in activating the carrier state. Therefore, our data indicate that the expression of *hns* is temperature-dependent, and highly expressed *hns* is a key host factor that maintains the carrier state lifestyle of HQ103 at 21°C.

### H-NS silences phage lytic promoter at 21°C to maintain a carrier state lifestyle

**Fig 4:**
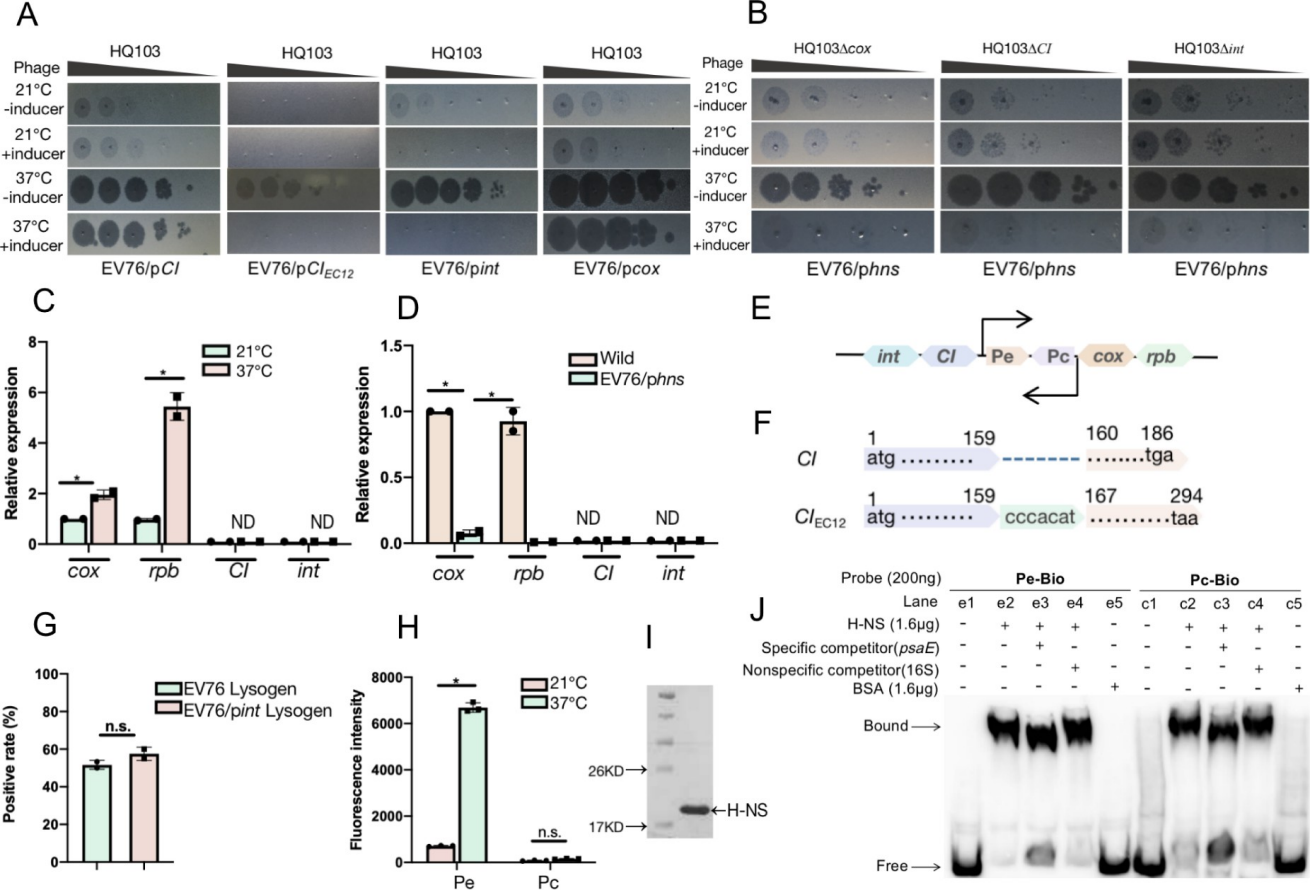
H-NS silences phage lytic promoter to maintain a carrier state lifestyle at 21°C. **(A)**The impact of *CI*, *int* and *cox* on the lytic-lysogenic switch. *CI*_*EC12*_ is a homologous *CI* encoded by the prophage of *E*.*coli* strain EC12. (**B**)Knock out of *CI*, *int* or *cox* in phage HQ103 does not affect the lytic-lysogenic switch. (**C**)The lytic promoter Pe controlled genes *cox* and *rpb* are 1.95±0.13 fold and 5.44±0.39 fold higher expressed at 37°C than at 21°C (*p*<0.05), while the Pc controlled gene *CI* and *int* are expressed at neither 37°C nor 21°C. (**D**)Overexpression of *hns* through plasmid could significantly inhibit the expression of *cox* and *rpb* down to 7.53±1.77% (*p*<0.01) and 0.97±0.12% (*p*<0.01) at 37°C when compared to the uninduced group. (**E**)The lytic- lysogenic switch elements of HQ103, including the lytic promoter Pe and lysogenic promoter Pc. (**F**)The sequence alignment of *CI* from HQ103 and *CI*_*EC12*_ indicates that the CI encoding gene of HQ103 has lost 7bp, and is truncated. (**G**) The percentage of colonies that contains the phage genome after re-streaking of EV76:: HQ103 or EV76/p*int*::HQ103 on the agar plates are 51.67±1.67% and 57.50±2.50%, respectively. (**H**)Reporter systems indicate that the lytic Pe promoter is more highly expressed at 37°C than at 21°C, and that the fluorescence of GFP is not detectable in the presence of Pc promoter. The asterisks mark *P*-value of < 0.05 as calculated by Student’s t-test, and n.s means no statistical difference. (**I**)Purification of H-NS protein. The protein marker and purified H-NS protein were separated on SDS-PAGE. (**J**)EMSA shows that H-NS binds to the promoter of both Pc and Pe. EMSA analyses were performed using the probes of Pe, Pc, a non-specific competitor probe (16s), and a specific competitor probe (psaE). The addition of H-NS protein, the probes, and BSA (negative control group) is indicated by ‘+’ sign and the omission is indicated by the ‘-’ sign.

The lytic-lysogenic switch of HQ103 (Fig 4E) is similar to that of *E*.*coli* phage P2. In P2, CI binds to the Pe promoter to repress *cox* expression and leads to lysogenization, while Cox binds to the Pc promoter to repress *CI* expression and promotes the lytic cycle [36].

To investigate how H-NS represses the expression of lytic genes, we cloned *CI*, *int*, and *cox* of HQ103 into the pBAD24 plasmid. The recombinant plasmids were then transferred into the EV76 strain and the cloned genes were induced by adding arabinose to the culture media. The results showed that EV76/p*CI* could not inhibit the lytic cycle at 37°C, and the sequence comparison revealed that the *CI* of HQ103 was truncated compared to its P2 counterparts and became non-functional due to a frameshift mutation caused by the loss of seven base pairs. In contrast, the overexpression of *CI*_*EC12*_ of a prophage encoded by *E*.*coli* strain EC12 could inhibit the formation of plaques.

Moreover, HQ103 could not form any plaque on EV76/p*int* at 37°C or 21°C (Fig 4A). The presence of phage genomes in the colonies at 21°C was confirmed by PCR using phage-specific primers. Approximately half of the colonies could maintain the presence of HQ103, the positive rate of which is similar to that of wild-type EV76 without a statistical difference (Fig 4G). Thus, this data indicates that integrase could inhibit the phage lytic cycle but could not promote the integration of HQ103 into EV76, probably because of the lack of an integration site in the genome of EV76.

Then, the expression of *CI* and *int* immediately after phage infection was measured by qRT-PCR, and the CT value of these two genes is non-detectable (Fig 4C). In contrast, *cox* and replication protein B gene (*rpb*) is highly expressed at 37°C but repressed at 21°C (Fig 4C). However, overexpression of *cox* could not promote the lytic lifestyle (Fig 4A). Because Cox inhibits the expression of *CI* and *int*, while neither CI nor Int could promote the carrier state.

The Pe promoter controls the expression of *cox* and other replication and lytic genes. Thus, we tested whether H-NS represses the expression of lytic genes by binding the Pe promoter. Reporter systems were constructed by inserting the Pe or Pc promoter region before GFP, and the fluorescence of GFP under the Pe promoter was 9.14-fold higher at 37°C than that at 21°C (Figs 4H, S2A), indicating significant repression of Pe at 21°C, while the fluorescence of GFP under the Pc promoter is not detectable at neither 21°C nor 37°C, confirming that Pc promoter is nonfunctional (Figs 4H, S2B). Moreover, when *hns* was overexpressed, the expression of *cox* and *rpb* decreased significantly to 7.53±1.77% (P<0.01) and 0.97±0.12% (P<0.01) at 37°C, respectively (Fig 4D), and the fluorescence of GFP under the Pe promoter was significantly repressed (S2C and S2D Fig). These data indicate that H-NS represses the Pe promoter.

Thus, we suspected that H-NS might directly bind to and inhibit the expression of both Pc and Pe. Therefore, *hns* from EV76 was cloned into *E*.*coli* BL21(DE3), and the protein was purified (Fig 4I). We further cloned Pc and Pe from HQ103, and EMSA shows that H-NS could efficiently bind both fragments (Fig 4J).

These data indicate the H-NS binds to the Pe promoter to inhibit the expression of lytic genes at 21°C, while the repression is weak at 37°C due to the downregulation of *hns*, which resulted in the expression of Pe to turn on the lytic cycle. *CI* and *int* are not expressed, and the expressed *cox* does not have any function because Cox represses *CI* and *int*, and neither of them is expressed. Thus, we knocked out *cox*, *CI*, and *int* in HQ103, and all three mutants had the same phenotype as wild-type phage HQ103 at 37°C or 21°C, and the lytic cycle could be inhibited by overexpression of *hns* for all three mutant phages (Fig 4B). These data indicate the carrier state lifestyle at 21°C is only due to the inhibition of Pe promoter by H-NS, but not the regulation of phage protein Cox, CI, or Int [36].

### Carrier state promotes phage HQ103 and *Y*. *pestis* coexistence in the soil at 21°C

**Fig 5:**
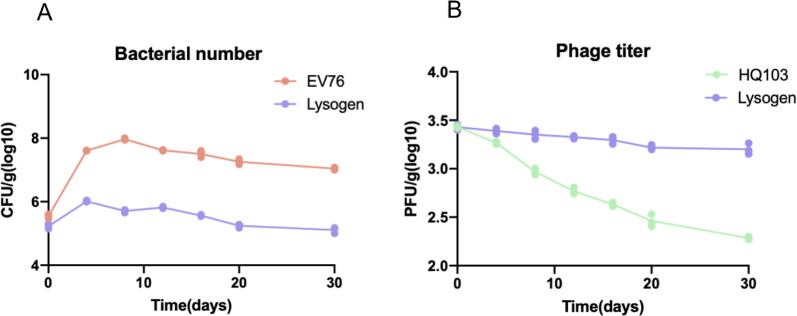
Carrier state promotes phage HQ103 and *Y*. *pestis* coexistence in the soil at 21°C. (A) Bacterial population dynamics of EV76 or phage carrier state EV76. (B) Phage or EV76::HQ103 was inoculated in the soil to monitor the changes in the phage titer. The CFU or PFU for each group was monitored for 30 days, and three biological repeats were performed.

Soil might be a reservoir of *Y*. *pestis* [10]. To test whether HQ103 could co-exist with *Y*. *pestis* in the soil at environmental temperature, phage HQ103, bacteria EV76, or carrier state bacteria EV76::HQ103 were inoculated into the soil and maintained for one month. The number of bacteria EV76 and carrier state bacteria grew for the first week and declined slowly probably due to the consumption of the nutrients in the soil (Fig 5A). On the contrary, when inoculating the phage HQ103 alone, the phage titer declined continuously from 2,696±49 PFU/g to 192±5 PFU/g on day 30, while for the carrier state bacteria inoculated soil, the phage titer was maintained for four weeks, and decreased from 2,690±134 PFU/g to 1,600±176 PFU/g on day 30 (Fig 5B). The stability of the phage titer in time indicated that the HQ103 phage is continuously released from a part of the lysed bacteria in the carrier state bacterial population. However, the phage carrier state may be costly for the bacteria because the number of the carrier state bacteria is much lower (Fig 5A). Thus, the carrier state promoted the coexistence of the phage and bacteria, which is beneficial to the maintenance of the phage population.

## Discussion

The evolution of phage genomes through mutations and recombination is a fascinating area of study [2,47]. The ability of phages to rapidly evolve allows them to adapt to changing environmental conditions and to overcome host defense mechanisms, which contributes to their success as biological entities. The emergence of a virulent phage from a temperate phage, as seen in the case of P2-like phage HQ103, is a striking example of the evolutionary plasticity of these viruses. This adaptation likely occurred in response to selective pressures in the environment, which favored the lytic lifestyle over the lysogenic one. For example, P2-like phages were isolated from diverse natural environments, defined as temperate phages with genomes ranging from 30 to 35 kb that infect γ-proteobacteria [48]. In this study, P2-like phage HQ103 had a truncated *CI* and a non-functional Pc promoter, so the lysogenic regulation elements are not functional and it could only turn on the lytic promoter and behaves like a lytic phage at 37°C, and lysis the host efficiently.

The phage lifestyle is classified as temperate or virulent [49], while the carrier state lifestyle is poorly described [5]. The definition of carrier state is still not well acknowledged, because of the limited studies in this field. A commonly referred definition of carrier state is that the phage was maintained through lytic infection of only a portion of the bacteria present [7,9]. However, the molecular mechanism that regulates the carrier state is still not well understood due to the lack of a stable phage-host model. The phage genes, such as *pid* of P22 phage [8], are required for maintaining the carrier state. However, a detailed molecular mechanism is not figured out yet.

We refer to HQ103 as a carrier state phage at 21°C based on two key pieces of evidence. Firstly, at 21°C, it could not efficiently lyse the host bacteria to initiate the lytic cycle or integrate into the genome of EV76 for stable lysogeny, while viral particle production was observed in EOP experiments. Secondly, approximately 50% of the progeny of lysogenic bacteria lost the phage during bacterial replication, indicating a non-symmetrical distribution of the phage genome. It’s important to note that in this system, phage HQ103 replicates less efficiently at 21°C, but the phage progenies are released from lysed bacteria in the carrier state. Thus, EV76::HQ103 is a novel carrier state model.

The potential ecological impact of the carrier state is being acknowledged. A phage genome inside the bacteria could promote horizontal gene transfer or recombination between different phages. Moreover, the carrier-state relationships could ensure the maintenance of both the bacteria and the phage under unfavorable conditions, such as nutrient-limited environments [5]. Herein, we found that the carrier state can maintain the presence of *Y*. *pestis* and phage in soil for a long time at environmental temperatures. However, both phage and *Y*. *pestis* died quickly under 37°C in soil because the soil dries quickly, making it difficult to draw any conclusion about the phage-host interactions at 37°C in soil (S3 Fig). Moreover, the phage titer continuously declines in the soil when viral particles were inoculated without bacteria but could maintain a relatively stable phage titer in the carrier state group. This indicates the constant release of the phage particle from a minority of the infected host, which is beneficial for the survival of the phage population (Fig 5). According to the classical perspectives, the lytic phage and host undergo rapid coevolution in test tubes or soils, characterized by arms race dynamics or fluctuating selection dynamics [35,50]. Thus, this study indicates that the carrier state could promote the phage-*Y*. *pestis* coexistence in soil.

**Fig 6:**
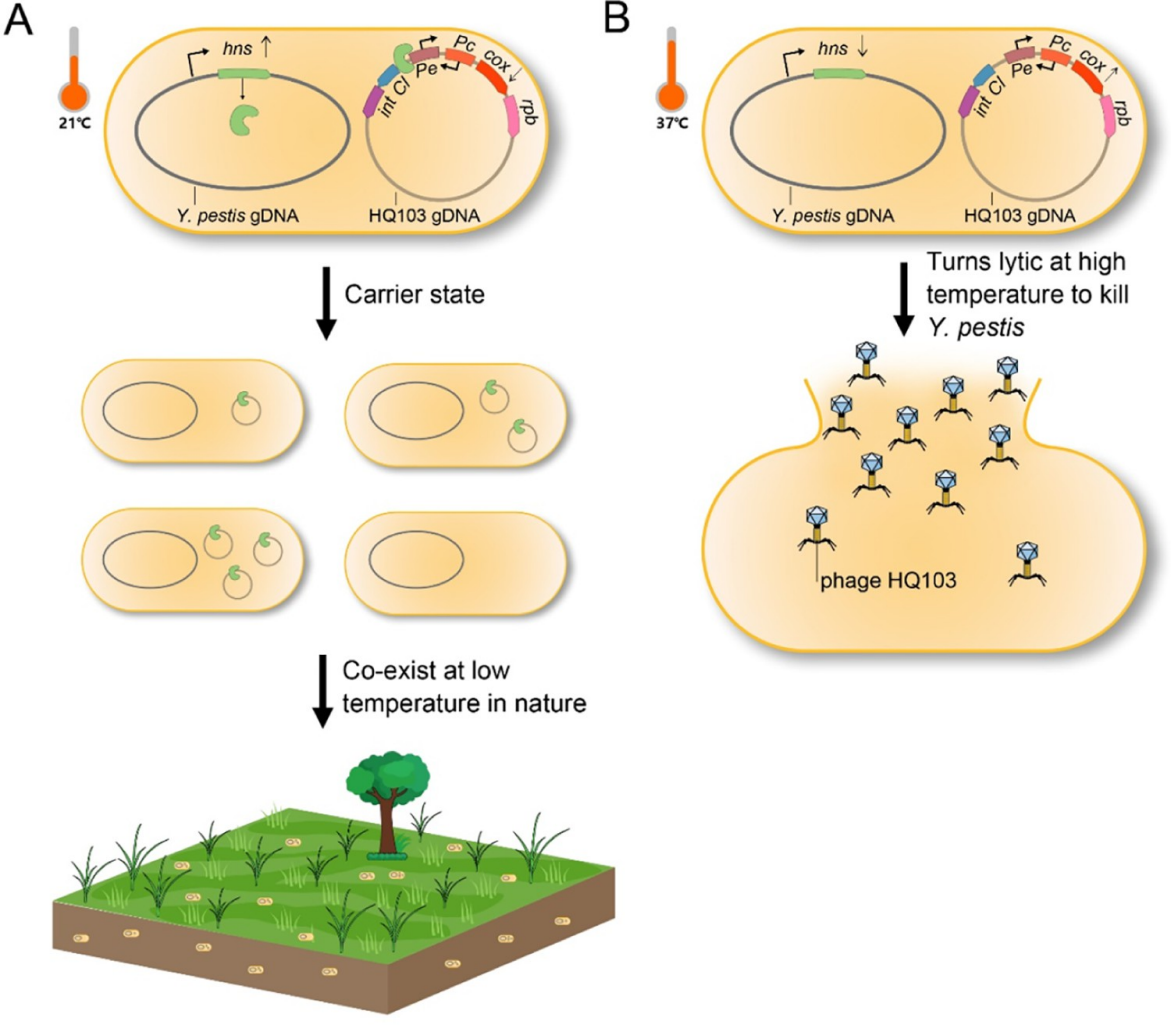
Model of the mechanism by which H-NS regulates the lytic-carrier state switch under different temperatures. (A) At 21°C, H-NS is highly expressed to bind to the Pe promoter to silence the lytic genes, leading to a carrier state lifestyle of HQ103, and promoting phage-bacteria coexistence in the soil. (B) At 37°C, H-NS is repressed and the Pe promoter is expressed, leading to the lytic cycle of HQ103 and the lysis of *Y*. *pestis*.

Meanwhile, H-NS is a well-known temperature-regulated silencing factor that regulates many host genes [41] and prophages [43]. For example, *Shewanella oneidensis* employs H-NS to repress prophage induction at high temperatures, but at low temperatures, H-NS is downregulated, leading to the promotion of P4-like cryptic prophage excision, biofilm formation, and the survival of the entire population [20]. However, the conversion of a virulent phage into a carrier state phage by H-NS has not been reported to the best of our knowledge (Fig 6).

In summary, the study of HQ103 reveals new functions of H-NS in phage biology beyond its canonical function of maintaining the integrated lysogeny. The HQ103 phage has a valid Pe promoter that controls the lytic cycles, which could be inhibited by H-NS. The study also provides a novel carrier state model for phage-host interactions, where the phage is not efficiently lysing the bacteria, nor integrating into the genome of the host, but rather being maintained as a minority inside the bacterial population. The carrier state could promote the coexistence of lytic phage and host in soil under environmental temperatures. These findings highlight the importance of studying the carrier state in phage biology and provide insights into the diverse mechanisms of phage-host interactions.

## Supporting information

S1 Table Host range of HQ103. (DOCX)

S2 Table Primers used in this study. (DOCX)

S3 Table DEGs detected by RNA-SEQ (excel file). (XLSX)

S1 Fig Illustration of the phage engineering protocol. To knock out *cox* in phage HQ103, the spacer was generated by annealing primers Δcox-G3F/R (S2 Table) and ligated into the Eco31I digested pPTCS. To construct the recombination template, primersΔcox-LA-F/R and Δcox-RA-F/R were used to amplify a 500 bp fragment before and after *cox*, respectively, and ligated into the Sam1/Xba1 digested plasmid by Gibson Assembly. Then, the plasmid was transferred into EV76. The strain EV76::pTCPLS-ΔcoxG3D was then infected with 10^5^ pfu of phages and used plaque assay to select the survivors. The mutant phage was verified by PCR followed by Sanger sequencing. Knock out of *CI*, *int* or *cox* in phage HQ103 was performed with similar technology with primers listed in S2 Table.
(TIF)

S2 Fig The microscopy images of the EV76 strains with the Pe or Pc promoter-GFP reporter plasmid. The Pe promoter is actively transcribed in EV76 at 37°C **(**A), while the Pc promoter is not transcribed at 37°C or 21°C (B). The overexpression of *hns* could significantly inhibit the expression of GFP under Pe promoter in EV76 at 37°C (C、D). The asterisks mark *P*-value of < 0.05 as calculated by Student’s t-test.
(TIF)

S3 Fig Phage HQ103 and *Y. pestis* died quickly in the soil at 37°C. (A) bacterial population dynamics of EV76 or phage carrier state EV76. (B) Phage or EV76::HQ103 was inoculated in the soil to monitor the changes in the phage titer. The CFU or PFU for each group was monitored and all of them died quickly probably because the soil dries quickly and making it difficult to draw any conclusion about the phage-host interactions in the soil at 37°C.
(TIF)
