# Supplementary figures and images for: Temperature-dependent carrier state mediated by H-NS promotes the long-term coexistence of Y. pestis and a phage in soil
Source: PLoS Pathog. 2023 Jun 22;19(6):e1011470. doi: 10.1371/journal.ppat.1011470 (PMC10321612; doi:10.1371/journal.ppat.1011470)

**S1 TABLE: Host range of phage HQ103**


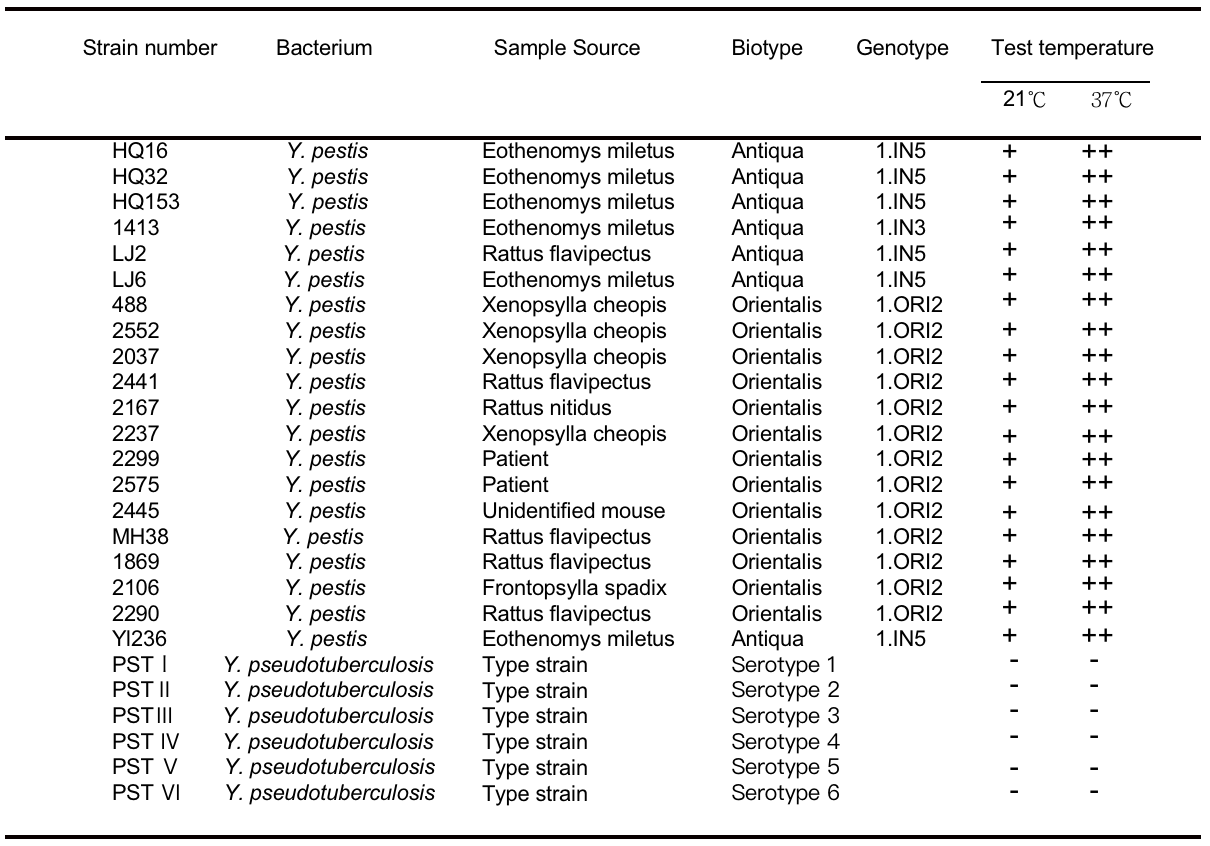
-: no plaque, +: small and blurred plaques, ++: large and clear plaques

Supplement: S1 Table — (DOCX) [file ppat.1011470.s001.docx]

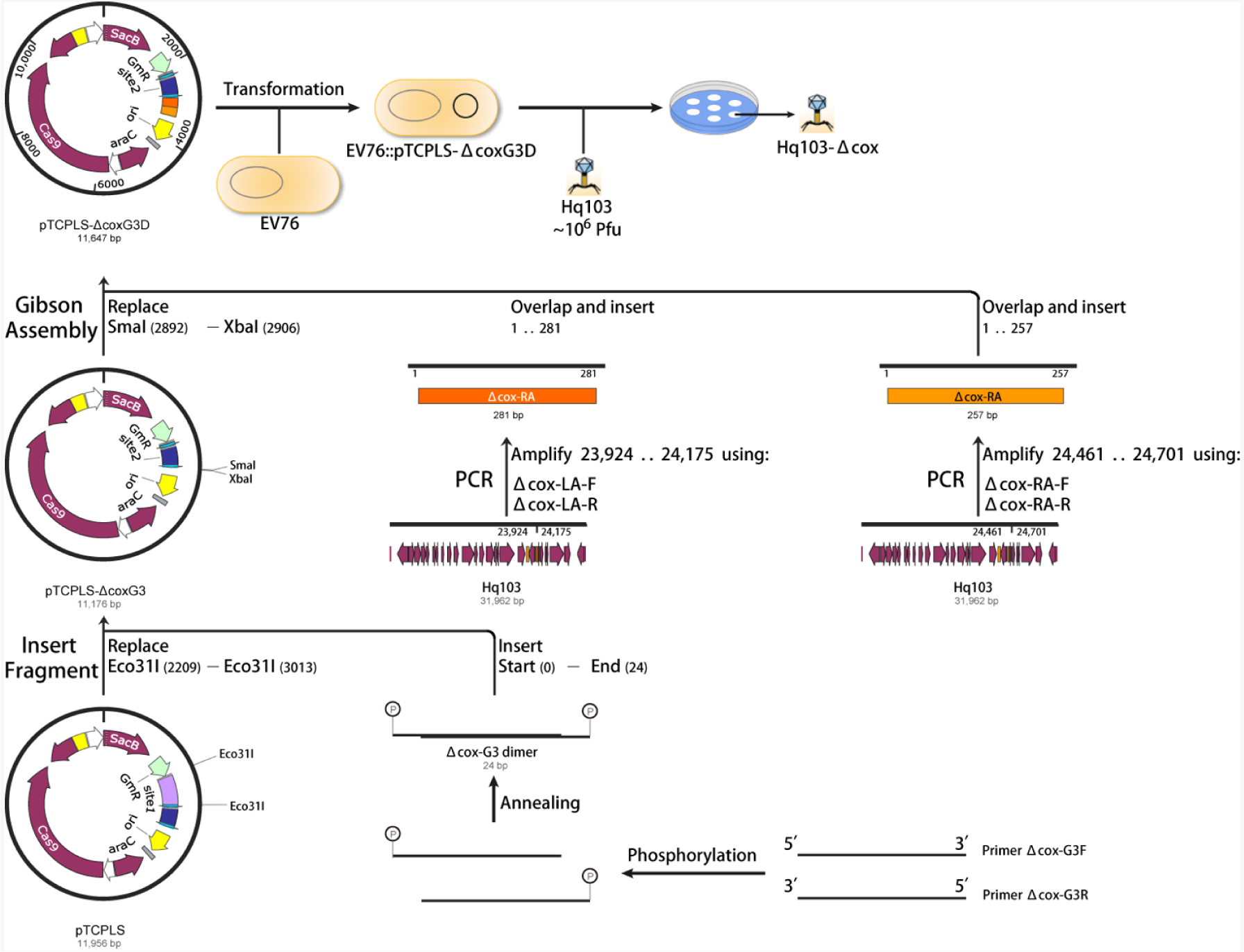

Supplement: S1 Fig — To knock out cox in phage HQ103, the spacer was generated by annealing primers Δcox-G3F/R (S2 Table) and ligated into the Eco31I digested pPTCS. To construct the recombination template, primersΔcox-LA-F/R and Δcox-RA-F/R were used to amplify a 500 bp fragment before and after cox, respectively, and ligated into the Sam1/Xba1 digested plasmid by Gibson Assembly. Then, the plasmid was transferred into EV76. The strain EV76::pTCPLS-ΔcoxG3D was then infected with 105 pfu of phages and used plaque assay to select the survivors. The mutant phage was verified by PCR followed by Sanger sequencing. Knock out of CI, int or cox in phage HQ103 was performed with similar technology with primers listed in S2 Table. (TIF) [file ppat.1011470.s004.tif]

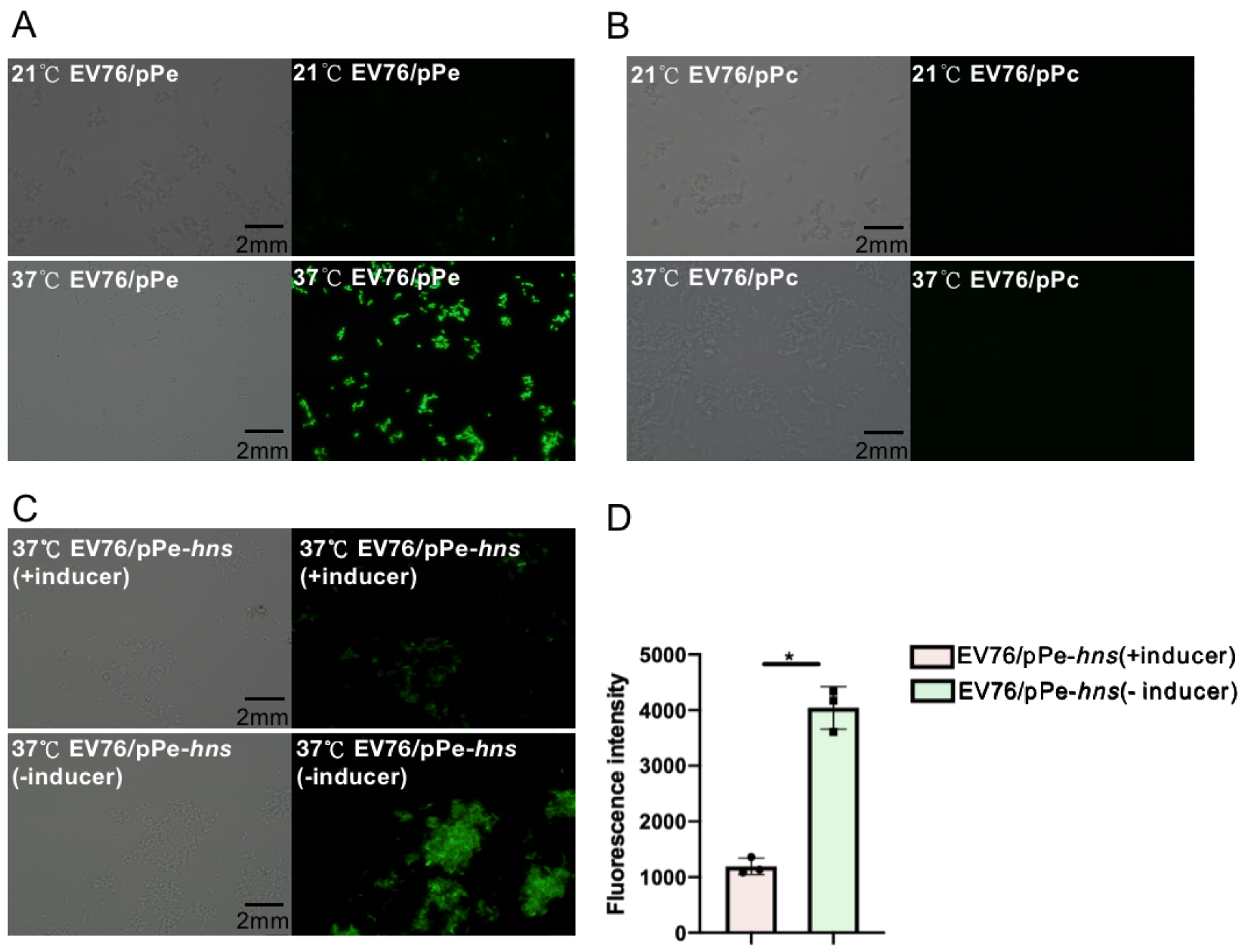

Supplement: S2 Fig — The Pe promoter is actively transcribed in EV76 at 37°C (A), while the Pc promoter is not transcribed at 37°C or 21°C (B). The overexpression of hns could significantly inhibit the expression of GFP under Pe promoter in EV76 at 37°C (C、D). The asterisks mark P-value of < 0.05 as calculated by Student’s t-test. (TIF) [file ppat.1011470.s005.tif]

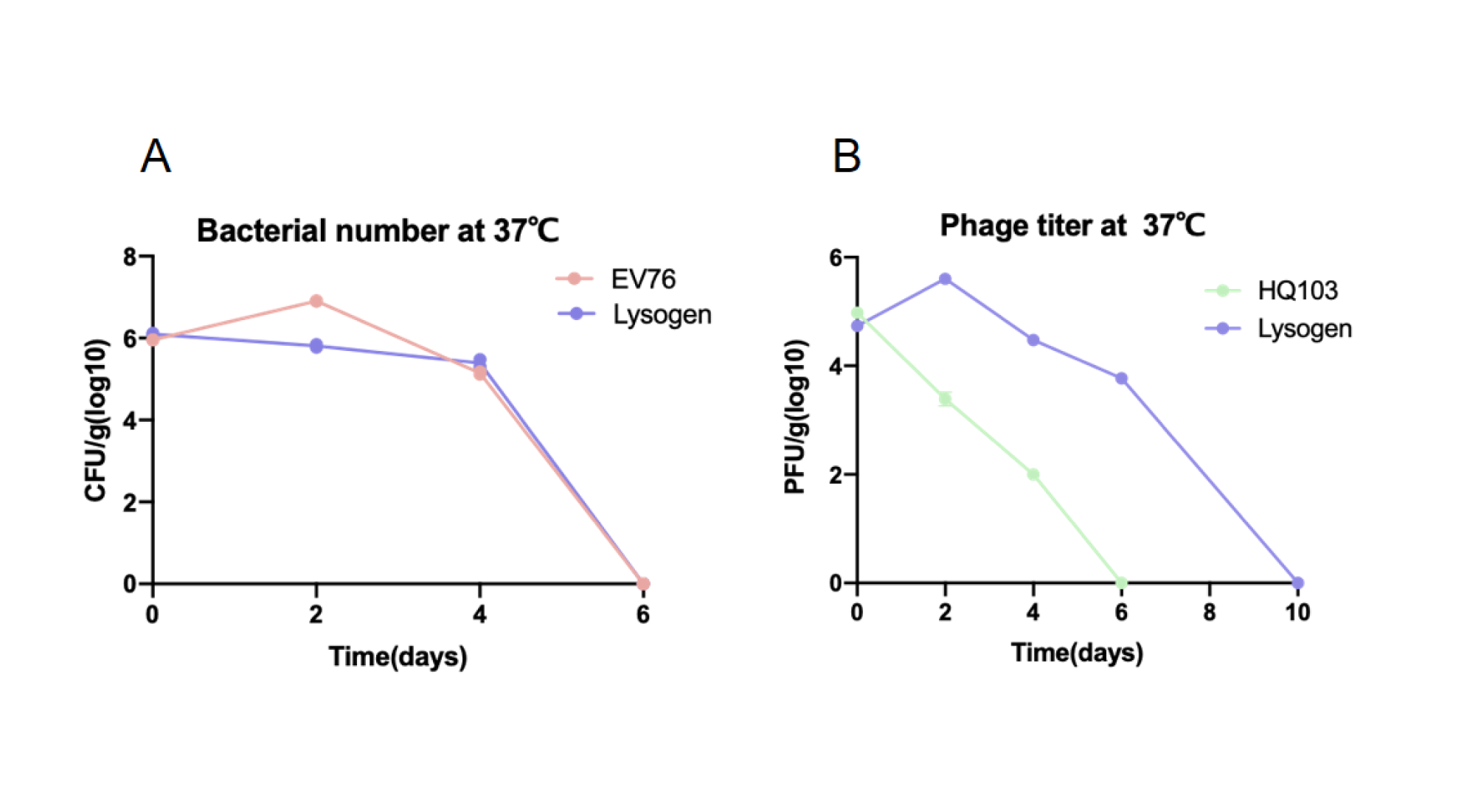

Supplement: S3 Fig — (A) bacterial population dynamics of EV76 or phage carrier state EV76. (B) Phage or EV76::HQ103 was inoculated in the soil to monitor the changes in the phage titer. The CFU or PFU for each group was monitored and all of them died quickly probably because the soil dries quickly and making it difficult to draw any conclusion about the phage-host interactions in the soil at 37°C. (TIF) [file ppat.1011470.s006.tif]
