# Supplementary material for: Temperature-dependent carrier state mediated by H-NS promotes the long-term coexistence of Y. pestis and a phage in soil
Source: PLoS Pathog. 2023 Jun 22;19(6):e1011470. doi: 10.1371/journal.ppat.1011470 (PMC10321612; doi:10.1371/journal.ppat.1011470)
Supplement: S2 Table — (DOCX) [file ppat.1011470.s002.docx]

**S2 TABLE: Primers used in this study**

| **Primers** | **Sequence (5’–3’)** |
| --- | --- |
| **qRT-PCR** |  |
| *recA* -Q-1F | GCACAGCGTGAAGGCAAAA |
| *recA* -Q-1R | CAACCGCACCAGAGCGAGTC |
| *lexA* -Q-1F | GCCCATTACGCACATCCTG |
| *lexA* -Q-1R | ACCCATCCCTGTTCAAGCC |
| 16s -Q-1F | TCCTCCTCGCTGAAAGTGC |
| 16s -Q-1R | ACGATCCCTAGCTGGTCTG |
| *hns*-Q-F | AGTGATTTGCCTTGCTCTT |
| *hns*-Q-R | GCTTATTGCTGACGGTATT |
| *int*-Q-F | GATGATGGTCGCTATGAAG |
| *int-*Q-R | CAGATGTCCAGCAACTCAC |
| *CI*-Q-1F | ATAGAGTTACCGCTAAGCAC |
| *CI* -Q-1R | TGTCATAAGCCATAGAGCA |
| *cox*-Q-1F | GCGATTAAACTCAGGAATG |
| *cox* -Q-1R | TTAGGGAAGCCAAAGACA |
| *rpb*-1F | GCAGATATTGCTCTGGTTTTAC |
| *rpb-*1R | GAATTCAATCAGCCGTACTGG |
| **Overexpression of**  **genes in EV76** |  |
| hns-gn-F1-EcoR1 | ccccGAATTC atgagcgaagcgttaaaaattc |
| hns-gn-R2-Xba1 | ccccTCTAGA ttacaacaggaagtcatccag |
| recA-gn-F-Nco1 | ccccCCATGG atggctattgatgagaat′ |
| recA-gn-R-Hind3 | ccccAAGCTT ttaaaattcttcgttggtttc |
| lexA-gn-F--EcoR1 | ccccGAATTC atgaaagcactaactaccagac |
| lexA-gn-R--Hind3 | ccccAAGCTT tcagatccagtccccgttacgga |
| Ch-*Cox*-F | GCTAGCAGGAGGAATTCACCatggacgctgaaaattatgtgattcag |
| Ch-*Cox*-R | CTTGCATGCCTGCAGGTCGAtcacaaccccatccacaaaagc |
| Ch-*Cox*-PB-F | ttttgtggatggggttgtgaTCGACCTGCAGGCATGC |
| Ch-*Cox*-PB-R | acataattttcagcgtccatGGTGAATTCCTCCTGCTAG |
| Ch-*CI*-F | GCTAGCAGGAGGAATTCACCatgaaattaagcgaaaagattaaggccttgc |
| Ch-*CI*-R | CTTGCATGCCTGCAGGTCGAttagccaatccttttcgcggag |
| Ch-*CI*-PB-F | ccgcgaaaaggattggctaaTCGACCTGCAGGCATGC |
| Ch-*CI*-PB-R | atcttttcgcttaatttcatGGTGAATTCCTCCTGCTAGC |
| Ch-*int*-F | GCTAGCAGGAGGAATTCACCatggcaattaagaagctcgatgatgg |
| Ch-*int*-F | CTTGCATGCCTGCAGGTCGAttataacgtcactccgccttttagtgg |
| Ch-*int*-PB-F | aaggcggagtgacgttataaTCGACCTGCAGGCATGC |
| Ch-*int*-PB-R | tcgagcttcttaattgccatGGTGAATTCCTCCTGCTAG |
| **H-NS purification** |  |
| *hns*-28F | TAAGAAGGAGATATACCATGAGCGAAGCGTTAAAAATTCT |
| *hns*-28R | TGGTGGTGGTGGTG CTCGAG CAACAGGAAGTCATCCAGT |
| **Reporter system** |  |
| RE-pe-F | gtcttcaagaattcgagctccttaatcttttcgcttaatttcatttgacatggt |
| RE-pe-R | GACATaattttccctccttatgagggaatttagtccctatataggcacc |
| RE-pe-P-F | atagggactaaattccctcataaggagggaaaattATGTCTAGAatgattaaaggag |
| RE-pe-P-R | aattaagcgaaaagattaaggagctcgaattcttgaagacgaaagg |
| RE-pc-F | gtcttcaagaattcgagctctaggatccagccctttctaaaccact |
| RE-pc-R | GACATaattttccctccttagatttgagaataaaccagcatctaaacggt |
| RE-pc-P-F | tgctggtttattctcaaatctaaggagggaaaattATGTCTAGAatgattaaaggagaaga |
| RE-pc-P-R | ttagaaagggctggatcctagagctcgaattcttgaagacgaaagg |
| HNS-GFP-pF | aattaagcgaaaagattaagAAGCTTGGCACTGGCC |
| HNS-GFP-pR | atttttaacgcttcgctcatCGTTTCACTCCATCCAAAAAAACGG |
| HNS-GFP-hF | TTTTTGGATGGAGTGAAACGatgagcgaagcgttaaaaattcttaacaaca |
| HNS-GFP-hR | tggatgagctctacaaataattacaacaggaagtcatccagtgatttg |
| HNS-GFP-gF | tggatgacttcctgttgtaattatttgtagagctcatccatgccatgt |
| HNS-GFP-gR | CGACGGCCAGTGCCAAGCTTcttaatcttttcgcttaatttcatttgacatggt |
| HNS-GFP-JCF1 | GCACGGCGTCACACTTTGC |
| HNS-GFP-JCF2 | gcaaatcactggatgacttcc |
| HNS-GFP-JCR2 | CTCTTCGCTATTACGCCAGC |
| **EMSA** |  |
| Pe-Bio F: | Bio-TGAGGGAATTTAGTCCCTATATAG |
| Pe-Bio R: | Bio-GATTTTTAGCAAAGCTGTGCCAC |
| Pc-Bio F: | Bio-TCACCATTTCACTGACGGC |
| Pc-Bio R: | Bio-GATTTGAGAATAAACCAGCATC |
| *psaE* F: | CCTGTTTGTCCTGCTGATCC |
| *psaE* R: | GACTCATTTGCCCTCACCTC |
| 16S F | TCCTCCTCGCTGAAAGTGC |
| 16S R | ACGATCCCTAGCTGGTCTG |
|  |  |
| **Identification of HQ103** |  |
| HQ103-1F | CGGTTTTAATGCAGTTGCATGAGT |
| HQ103-1R | CTTGGATTTTCACATAGTGTCGGA |
| **Knock out phage genes** |  |
| Spacer construction |  |
| ΔCI-G1F | TAGTagtccaaccactccgcgaaa |
| ΔCI-G1R | AAACtttcgcggagtggttggact |
| Δcox-G3F | TAGTaataactgagcaatacaacg |
| Δcox-G3R | AAACcgttgtattgctcagttatt |
| Δint-G1F | TAGTgctctgaatccactaaaagg |
| Δint-G1R | AAACccttttagtggattcagagc |
| Donor construction |  |
| ΔCI-LA-F | TTTTTTTGGCGCGCCttcaccatttcactgac |
| ΔCI-LA-R | gaaaatgtaatctttatatagagttgacatggtgcctatataggg |
| ΔCI-RA-F | ctctatataaagattacattttcaccatttgc |
| ΔCI-RA-R | GGTCTGACAGCTCGAGcaactcactcaaagtccgg |
| Δcox-LA-F | TTTTTTTGGCGCGCCgggttcaaagtttcctcc |
| Δcox-LA-R | cgcgCctcattcataacataggatccagccctttc |
| Δcox-RA-F | ttatgaatgagGcgcgttgtattg |
| Δcox-RA-R | GGTCTGACAGCTCGAGaagatgcttgccaattatcc |
| Δint-LA-F | TTTTTTTGGCGCGCCttcaccatttcactgac |
| Δint-LA-R | actcagaggggggaaatttagccaatTcttttcgcgg |
| Δint-RA-R | GGTCTGACAGCTCGAGacgtgaattgcagtgcc |
| Δint-LA-R | actcagaggggggaaatttagccaatTcttttcgcgg |
